# Upregulation of *Lactobacillus spp*. in gut microbiota as a novel mechanism for environmental eustress-induced anti-pancreatic cancer effects

**DOI:** 10.1080/19490976.2025.2470372

**Published:** 2025-02-23

**Authors:** Yiyi Liang, Min Du, Xin Li, Jian Gao, Qian Li, Huimin Li, Jin Li, Xiang Gao, Hui Cong, Yimeng Huang, Xinran Li, Liwei Wang, Jiujie Cui, Yu Gan, Hong Tu

**Affiliations:** a State Key Laboratory of Systems Medicine for Cancer, Shanghai Cancer Institute, Renji Hospital, Shanghai Jiao Tong University School of Medicine, Shanghai, China; b School of Basic Medicine, Fudan University, Shanghai, China; c Department of Oncology, Renji Hospital, Shanghai Jiao Tong University School of Medicine, Shanghai, China

**Keywords:** Psychological eustress, pancreatic ductal adenocarcinoma (PDAC), gut microbiota, *Lactobacillus spp*.

## Abstract

Pancreatic ductal adenocarcinoma (PDAC) is a highly lethal malignancy with limited effective treatment options. Emerging evidence links enriched environment (EE)-induced eustress to PDAC inhibition. However, the underlying mechanisms remain unclear. In this study, we explored the role of gut microbiota in PDAC-suppressive effects of EE. We demonstrated that depletion of gut microbiota with antibiotics abolished EE-induced tumor suppression, while fecal microbiota transplantation (FMT) from EE mice significantly inhibited tumor growth in both subcutaneous and orthotopic PDAC models housed in standard environment. 16S rRNA sequencing revealed that EE enhanced gut microbiota diversity and selectively enriched probiotic *Lactobacillus*, particularly *L. reuteri*. Treatment with *L. reuteri* significantly suppressed PDAC tumor growth and increased natural killer (NK) cell infiltration into the tumor microenvironment. Depletion of NK cells alleviated the anti-tumor effects of *L. reuteri*, underscoring the essential role of NK cell-mediated immunity in anti-tumor response. Clinical analysis of PDAC patients showed that higher fecal *Lactobacillus* abundance correlated with improved progression-free and overall survival, further supporting the therapeutic potential of *L. reuteri* in PDAC. Overall, this study identifies gut microbiota as a systemic regulator of PDAC under psychological stress. Supplementation of psychobiotic *Lactobacillus* may offer a novel therapeutic strategy for PDAC.

## Introduction

The incidence of pancreatic ductal adenocarcinoma (PDAC) is rising globally, posing a substantial disease burden and high mortality rates. Most patients are diagnosed at the advanced stage, leading to a dismal prognosis with a 5-year relative survival rate of only 13%.^1^ PDAC exhibits resistance to various therapeutic approaches, including immune checkpoint blockades (ICB), thus rendering the prognosis for PDAC patients grim despite decades of progress in cancer treatment.

Psychological distress has long been suspected to influence cancer risk and mortality.^2,3^ Moreover, recent studies have demonstrated that emotional distress can affect the therapeutic efficacy of ICB in melanoma^4^ and non-small-cell lung cancer.^5^ With regard to PDAC, according to a case-control study nested within the UK Biobank cohort, distress-related factors and stressful events play a negative role in susceptibility to PDAC independent of genetic background.^6^ Psychological distress is also related to PDAC mortality. Based on the pooling data from 16 prospective cohort studies, hazard ratios (HR) for psychological distress in relation to PDAC death outcome was 2.76 (95% confidence interval (CI): 1.47–5.19) after adjusted with age, sex, BMI, educational attainment, smoking status and alcohol consumption.^7^ A population-based study revealed that depression and pancreatic cancer are closely associated (odds ratio (OR): 2.4, 95% CI: 1.15–4.78).^8^ It is worth noting that depression more frequently precedes PDAC than it does for other gastrointestinal malignancies (OR: 4.6, CI: 1.07–19.4) or all types of cancer (OR: 4.1, CI: 1.05–16.0).^8^ These epidemiological studies highlight the urgent need to investigate how the brain regulates the growth of peripheral cancers, with a particular focus on pancreatic cancer.

Animal studies have demonstrated that exposure to distress environments such as social defeat,^9^ social isolation^10^ and chronic restraint stress^11^ substantially facilitate cancer growth and compromise anti-tumor immunity.^12^ On the contrary, dwelling in an enriched environment (EE), a complex rearing setting characterized by more space, physical activity and social interactions, is capable to inhibit the growth of multiple tumors in murine tumor models.^13,14^ Previously, we reported that EE-induced eustress displayed a potent anti-PDAC phenotype.^15–17^ A brain-adipocyte (BDNF/Leptin) axis has been proposed to explain the EE-induced anti-tumor property in mice models of melanoma and colon cancer.^13^ It is also reported by us and several groups that EE promotes the maturation, proliferation and tumor infiltration of immune cells including natural killer (NK),^15,18,19^ T^20,21^ and B cells.^22^ Moreover, we reported that EE suppresses cancer cell proliferation through downregulating energy metabolism.^23^ Though EE was found to enhance gut microbiota biodiversity in a mouse model of colon cancer,^24^ it remains unclear whether the EE-induced changes in gut microbiota composition are responsible for the anti-tumor effect. It is also unknown which specific bacteria EE-induced to make this effect.

Polymorphic microbiomes have emerged as one of the hallmarks of cancer.^25^ Patients with PDAC have a distinct gut microbial profile, characterized by an increase in possible pathogens and a decrease in specific probiotics such as butyrate-producing bacteria.^26^ Alterations in the microbiota composition in fecal^27–29^ and tumor tissues^30–32^ are associated with the carcinogenesis, progression and treatment response in PDAC. In murine models, microbes such as *Bifidobacterium pseudolongum* has been shown to induce carcinogenesis in the pancreatic duct,^33^ supporting a possible role of gut microbiota in the etiology of PDAC. Given the important function of microbiota in the development of PDAC, it is reasonable to speculate that probiotic supplementation may have potential as part of an integrative therapy for PDAC. However, to date, there are limited probiotic candidates that have demonstrated therapeutic efficacy in preclinical studies, and no clinical trials have evaluated probiotics as an independent therapeutic approach for PDAC.

The importance of the gut-brain axis in maintaining homeostasis has long been recognized.^34^ The gut microbiota has emerged as a key regulator of gut-brain signaling, influencing both enteric and central nervous system functions.^35^ On the one hand, mental and psychological factors influence gut microbiota composition; on the other hand, certain bacteria can affect anxiety- and stress-related behaviors. Thus, it is interesting to know whether this bidirectional regulatory effect of the gut microbiota plays a role in the systemic regulation of cancers. Here, we report that the tumor-suppressive effects of EE can be transferred to the SE mice through fecal transplantation, indicating the brain-gut-microbiota axis is indeed involved. Additionally, we identify a commensal microbial species *Lactobacillus reuteri* (*L. reuteri*) as an EE-sensitive probiotic with anti-PDAC properties. Our findings highlight the important role of gut microbiota in the interplay between psychological eustress and tumor resistance, suggesting that manipulating gut microbiome by using psychobiotics may provide a novel therapeutic strategy for PDAC.

## Materials and methods

### Cell line

The Panc02 murine pancreatic cancer cells were obtained from Frederick National Laboratory and cultured in DMEM (#SH30022, HyClone, USA) supplemented with 10% FBS (#10099141, Gibco, USA) and 1% penicillin-streptomycin (#SV30010, HyClone, USA).

### Mice

All animal studies were approved by the Animal Care and Use Committees of Shanghai Cancer Institute and were manipulated by the institutional guidelines. Male C57BL/6 mice, purchased from Sino-British SIPPR/BK Lab Animal Ltd (Shanghai, China), were used in murine experiments. For the subcutaneous tumor model, 6-week-old male mice were subcutaneously injected with 1 × 10^6^ Panc02 cells (per mouse) in the right armpit. For the orthotopic tumor model, a small left-side abdominal incision near the spleen was made, and the pancreas was found and identified in front of the right side of the spleen.^36^ Five hundred thousand Panc02 cells were injected into the pancreas using a sterile insulin needle.

For the enriched environment (EE) experiments, 3-week-old mice were randomly assigned to either EE or standard environment (SE) conditions for 3 weeks. SE and EE were established as previously described.^16^ Following this period, they were subcutaneously implanted with Panc02 cells. Tumors at different times were identified and measured every 3-4 days as previously described.^15^ At the time of endpoint, the tumor and tissues of each mouse were harvested and processed for analysis.

### Antibiotic treatment

To ablate the gut microbiome, 4–6 weeks old male mice were administered an antibiotic cocktail (Abx), which contained 1 g/L ampicillin (#M2390, abmole, USA), 0.5 g/L metronidazole (#M3311, abmole, USA) and 1 g/L neomycin (#M3594, abmole, USA), in their daily drinking water.

### Fecal microbiota transplantation (FMT)

After being housed under SE or EE conditions for 3 weeks, fresh fecal samples of mice were collected. SE or EE mouse fecal bacteria suspension was obtained and preserved at -80°C ultra-low temperature freezer. The FMT treatment was performed as previously described.^37^ Briefly, the mice were treated with Abx to eliminate intestinal bacteria before FMT. Fecal suspension of SE or EE mice was transplanted into 5-6 weeks-old male mice once every 3 days by oral gavage (o.g.). After one week of FMT treatment, Panc02 cells were injected into mice. The tumor-bearing mice were treated with FMT twice a week for 4 weeks or 2 weeks during which the subcutaneous or orthotopic tumor growth was monitored.

### FITC-dextran permeability assay

FITC-dextran (#46944, Sigma, USA; 400 mg/kg, 4 kD) was orally administered to mice after food and water fasting for 4 h. Serum samples were obtained 4 h after FITC-dextran administration. The serum fluorescence intensity was measured with a spectrophotometer at an excitation wavelength of 490 nm and an emission wavelength of 530 nm.

### Culture of *Lactobacillus reuteri* (*L.reuteri*) and *Lactobacillus johnsonii* (*L.johnsonii*)

*L. reuteri* (ATCC PTA 6475) and *L. johnsonii* (CGMCC 1.3255) were cultured in Mann Rogosa Sharpe (MRS) broth medium (#HB0384, Haibo media, Qingdao, China) in anaerobic conditions (DG250, Don Whitley Scientific, West Yorkshire, UK). The amount of bacteria was measured by the absorbance at 600 nm with an OD value of 1.6-1.8.

### Probiotics treatment

For the *Lactobacillus* cocktail experiment, 6-week-old male mice were treated with Abx for 2 weeks and received an injection of Panc02 cells. Then, the tumor-bearing mice of *Lactobacillus* cocktail group were administrated with 2.5 × 10^8^ CFU of *L. reuteri* and *L. johnsonii* in 100 µl PBS per mouse by o.g. twice a week for 4 weeks. At the same time, control mice were given an equivalent volume of PBS. For the *Lactobacillus* strains selection experiment in the subcutaneous tumor model, mice in the treated group were randomly assigned to the following 2 groups: (1) *L. reuteri* group: 2.5 × 10^8^ CFU/mouse; (2) *L. johnsonii* group: 2.5 × 10^8^ CFU/mouse. For the *L. reuteri* experiment in the orthotopic tumor model, mice were administrated with 2.5 × 10^8^ CFU/mouse of *L. reuteri* twice a week for 4 weeks.

### Anxiety-like behaviors test

The anxiety-like behaviors were examined using an elevated plus maze (EPM) test as the publication described.^38^ To monitor the effectiveness of EE housing, mice exposed to SE or EE conditions for 3 weeks were subjected to EPM test. For the probiotics experiment, following 3 weeks of treatment of probiotic administration, the mice were individually placed for EPM test. The test session of each mouse was recorded and then analyzed using SuperMaze software (version 2.0; Shanghai Xinruan Information Tech Co., Shanghai, China).

### Anti-NK1.1 treatment in mice

For anti-NK1.1 treatment, mice were treated with 90 μg anti-NK1.1 (#BE0036, clone PK136, Bio X Cell, USA) antibodies or isotype control (#BE0085, clone C1.18.4, Bio X Cell, USA) beginning on day 1 before tumor engraftment and on days 1, 4, 7, 14, 21 after Panc02 cells inoculation. NK depletion was confirmed by flow cytometry with the absence of CD3^−^NK1.1^+^ cells in the spleen.

### Flow cytometry

The spleen and tumor cells were prepared as our previously described.^15^ Then, the cells were stained with anti-mouse CD3-PerCP-Cy5.5 (#100218, clone: 145-2C11, Biolegend, USA), CD3-APC (#100311, clone: 145-2C11, Biolegend, USA), NK1.1-FITC (#11-5941, clone: PK136, eBioscience, USA), NK1.1-PE (#50-5941, clone: PK136, Tonbo, USA), CD8-APC (#20-0081, clone:53-6.7, Tonbo, USA) and CD19-PE (#115507, clone:6D5, Biolegend, USA) antibodies and the appropriate isotype controls following incubation with anti-CD16/CD32 antibodies (#14-0161, clone: 93, eBioscience, USA) to block nonspecific and Fc-mediated binding. The stained cells were detected using a FACSCelesta flow cytometer (BD Biosciences, USA) and analyzed using FlowJo™10 software (BD Biosciences, USA).

### Histochemical and immunohistochemical staining

For IHC analysis, the tumor tissue slides were incubated with polyclonal antibodies against ZO-1 (#21773-1-AP, Proteintech, USA; dilution 1:2000), MUC2 (#ab272692, Abcam, UK; dilution 1:200), Ki67 (#ab15580, Abcam, UK; dilution 1:200) and NK1.1 (#ab197979, Abcam, UK; dilution 1:200). Ten randomly selected fields were examined for each sample. For details about immunohistochemical staining, see our previous publication.^15^ For periodic acid-Schiff (PAS) staining, Schiff’s reagent and hematoxylin solution were used (Ruiyu Tech Co., Shanghai, China). The detailed procedures were processed as previously described.^39^

To quantify goblet cells, the number of positive cells per 20× microscope field was counted (10 random fields per mouse). The intensity of ZO-1 was effectively quantified using the H-score evaluation. The H-score is a reliable metric calculated as follows: (1×percentage of weak staining) + (2×percentage of moderate staining) + (3×percentage of strong staining) within the target region. For the quantification of Ki67, the percentage of nuclei with positive staining in tumor per 1000 tumor cells was scored. To quantify NK1.1^+^ cells, the number of positive cells per 20× microscope field was counted (10 random fields in the tumor tissue area). Scoring was independently conducted by two pathologists blinded to the clinical parameters.

### Disbiome database analysis

The relative abundance of key taxa in PDAC and depression was obtained using Disbiome (https://disbiome.ugent.be/hom).

### Fecal bacterial DNA extraction and bacterial identification

For bacterial DNA identification, mice feces DNA was isolated using the E.Z.N.A. Bacterial DNA Kit (#D3350, Omega, USA) according to the manufacturer’s protocol. All amplifications were performed using the StepOne Plus Real-time PCR System (Applied Biosystems, USA) with SYBR Green reagents (#04887352001, Roche Diagnostics, Switzerland). The average cycle threshold (Ct) value was calculated from triplicates. The relative abundance of *L. reuteri* based on the ΔCt value was defined as Ct (*L. reuteri*) -Ct (16S ribosome genes). The Ct value for any sample not amplified after 40 cycles was defined as 40 (threshold of detection). The fold difference of relative abundance was calculated by 2^−ΔCt^.

Primers for the quantitative real-time PCR:

16S-Forward: AGAGTTTGATCCTGGCTCAG

16S-Reverse: ATTACCGCGGCTGCTGG

*L. reuteri*-Forward: TGAATTGACGATGGATCACCAGTG

*L. reuteri*-Reverse: CGACGACCATGAACCACCTGT

*L. johnsonii*-Forward: CACTAGACGCATGTCTAGAG

*L. johnsonii*-Reverse: AGTCTCTCAACTCGGCTATG

### 16S rRNA sequencing and analysis

Sequencing analysis of bacterial 16S rRNA was performed to analyze the microbiota composition in feces and stools. Total genomic DNA from feces and stool was extracted using the CTAB method. The hypervariable V3-V4 regions of 16S rRNA genes were amplified using the specific primer pair 341F (5'-CCTAYGGGRBGCASCAG-3') and 806 R (5'-GGACTACNNG GGTATCTAAT-3'). Sequencing was performed at the Illumina NovaSeq platform (Illumina MiSeq, USA) by Wekomo Technology Company (Shenzhen, China). Sequencing data processing and bioinformatics analysis were conducted on the Bioincloud Platform (https://www.bioincloud.tech/).

### Clinical samples and study cohorts

Two cohorts of PDAC patients from Renji Hospital affiliated with Shanghai Jiao Tong University School of Medicine were involved between 2022 and 2024 (KY2020-188-07-02). Informed consent was obtained from all patients.

In cohort 1, 30 PDAC patients (stage IIIB-IV) who are about to receive drug therapy are screened for enrollment in this study. Emotional distress (ED) is assessed using the SDS and SAS scales. Stool samples were collected from PDAC patients before receiving therapy and stored at -80°C immediately. Patients were classified as having no ED if their scores on the SDS and SAS scales were below 39, while those with scores at or above 39 were classified as experiencing ED. We confirmed the therapy response based on RECIST v1.1 criteria. We performed 16S rRNA sequencing on stool samples to assess which bacterium is predominant (and/or different) in the no ED group compared with the ED group. The clinical characteristics of this study participants are shown in Table S1.

In cohort 2, clinical features and stool samples from 33 PDAC patients were collected. Patients were classified as low and high *Lactobacillus* groups based on the relative abundance in stool. Survival visits were performed every 3 months. Progression-free survival (PFS) was defined as the duration between the date of initiation of data collection and disease progression or death, whichever occurred first. Overall survival (OS) refers to the duration between the date of initiation of diagnosis and death from any cause. The data cutoff was 15 June 2024. We compared the circulating NK cell counts and percentage in serum samples between groups. The clinical characteristics of this study participants are shown in Table S2.

### Data statistical analysis

Data were examined to determine whether they were normally distributed with the one-sample Kolmogorov-Smirnov (K-S) test. All normally distributed data were presented as means ± standard error of the mean (SEM), and comparisons of measurement data between two groups were performed using independent sample Student’s *t*-test, and comparisons among three or more groups were performed by one-way ANOVA. If the K-S results showed significant differences, when data were skewed, comparisons were performed by the nonparametric Mann-Whitney test. PFS and OS were calculated using a Kaplan-Meier survival curve, and the log-rank test was used for comparison. *DESeq2* was used to evaluate the statistically significant relative abundance of specific microbes between groups at the species level. The Kruskal-Wallis test was applied to determine the statistical significance of differences in gut microbial diversity between groups. The statistical analysis was performed with GraphPad Prism 9.0 (GraphPad Software Ltd, USA). A *p*-value <0.05 was considered statistically significant and a *p*-value ≥0.05 was considered statistically no significant (ns).

## Results

### Gut microbiota mediates the eustress-induced tumor-suppressive effect in pancreatic cancer

**Figure 1.**
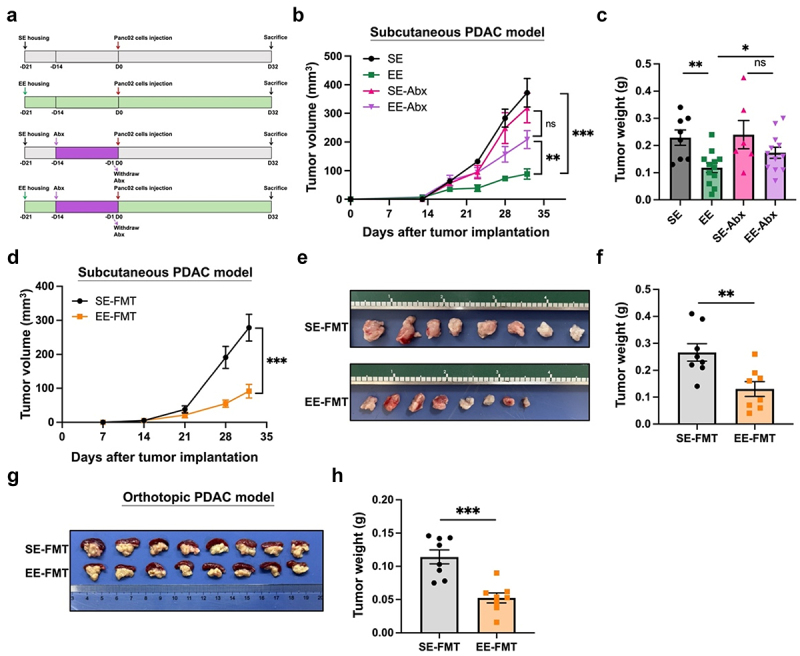
Gut microbiota mediates the EE-induced anti-PDAC effect in mice. (a) Schematic diagram of Abx treatment in SE and EE tumor-bearing mice (*n* = 6-8 for SE and SE+Abx groups, *n* = 12 for EE and EE+Abx groups). Abx treatment significantly diminished the EE inhibitory effect of Panc02 tumor growth, as evidenced by (b) tumor volume and (c) tumor weight. Abx-treated C57BL/6 mice received FMT and then Panc02 cell implantation. The transplantation of feces from EE mice to SE mice markedly retarded tumor growth, as supported by (d) tumor volume, (e) tumor images, (f) tumor weight in the subcutaneous PDAC model and (g) tumor images, (h) tumor weight in the orthotopic PDAC model (*n* = 8/group). ***, *p* < 0.001, **, *p* < 0.01; *, *p* < 0.05; ns, not significant.

To assess whether gut microbiota was involved in EE-induced tumor inhibition, we used the antibiotic cocktail (Abx) to deplete the gut microbiota before tumor implantation (Figure 1(a)). As expected, in the absence of Abx, EE led to a significant inhibition of tumor growth (Figure 1(b)). At the time of sacrifice, the tumor weight of EE group was significantly lower than that of SE group (SE group: 0.23 ± 0.03 g *vs*. EE group: 0.12 ± 0.02 g, *p* < 0.01, Figure 1(c)). However, when mice were treated with Abx, there was no significant difference in the volume and weight (SE-Abx group: 0.24 ± 0.05 g *vs*. EE-Abx group: 0.17 ± 0.02 g, *p* = 0.1646, Figure 1(b–c)) of subcutaneous tumors between the EE and SE groups, indicating that Abx treatment abolished the anti-PDAC effect of EE and gut microbiota is involved in EE-induced tumor inhibition.

To further confirm this notion, we next conducted a fecal microbiota transplantation (FMT) experiment using fecal samples from different donor mice: those housed in EE and those housed in SE. The recipient mice receiving feces from EE donors (EE-FMT) or feces from SE donors (SE-FMT) were transplanted with Panc02 cells and maintained under the same standard rearing conditions throughout the entire experimental procedure. In a subcutaneous tumor model of PDAC, the EE-FMT group exhibited significantly slower tumor growth compared to the SE-FMT group (Figure 1(d)). At the end of the experiment (32 days post-tumor implantation), the average tumor weight in the EE-FMT group was 0.13 ± 0.03 g, markedly lower than the 0.27 ± 0.03 g observed in the SE-FMT group (*p* < 0.01, Figure 1(e–f)). Consistent findings were noted in the orthotopic PDAC model, where the EE-FMT group demonstrated a reduction in average tumor weight by approximately 55% compared to the SE-FMT group (SE-FMT group: 0.11 ± 0.01 g *vs*. EE-FMT group: 0.05 ± 0.01 g, *p* < 0.001, Figure 1(g–h)).

### Eustress restores gut microbiota diversity and strengthens the intestinal barrier in pancreatic cancer-bearing mice

**Figure 2.**
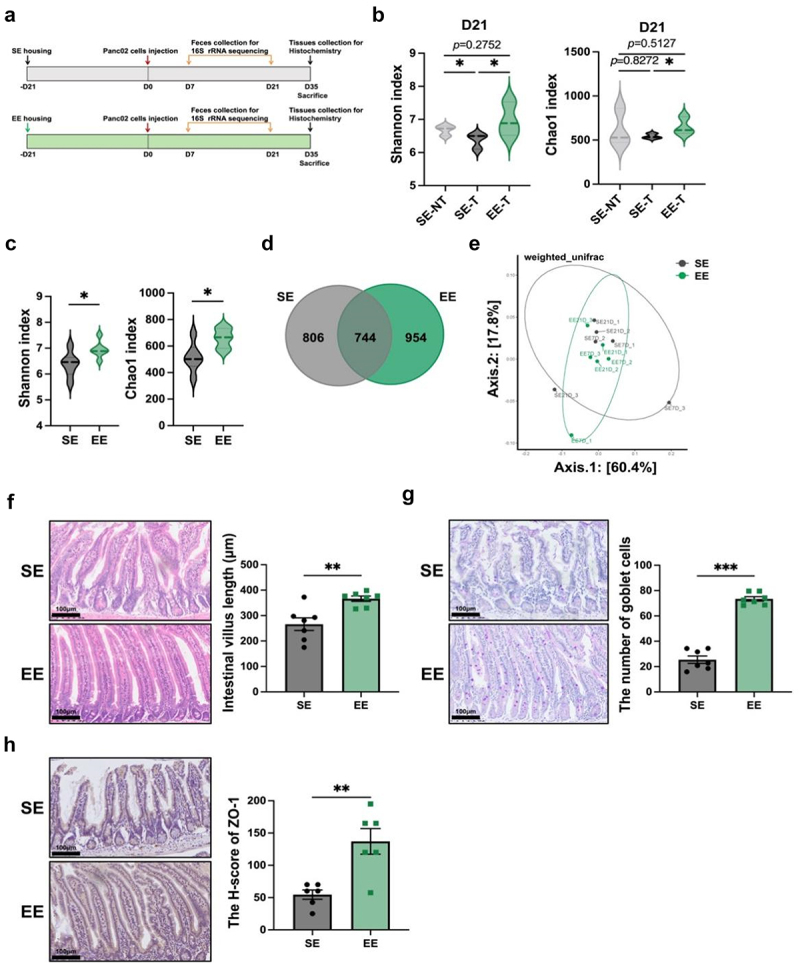
Eustress restores gut microbiota diversity and strengthens the intestinal barrier in pancreatic cancer-bearing mice. (a) Schematic diagram of gut barrier function analysis in SE and EE tumor-bearing mice using 16S rRNA sequencing and histochemistry. PDAC destroyed gut homeostasis, as shown by (b) α diversity analysis of gut microbiome including Chao1 and Shannon indexes in feces by 16S rRNA sequencing. Compared with SE tumor-bearing mice, EE mice exhibited increased phylogenetic diversity and richness in fecal samples post-PDAC implantation (day 7 and 21 pool), as supported by (c) Chao1 and Shannon indexes, (d) the Venn diagram analysis of the shared and unique genera and (e) β diversity analysis based on weighted UniFrac metrics using Principal coordinate analysis (PCoA). Histochemical staining confirmed that EE preserved gut barrier function in tumor-bearing mice, as evidenced by (f) representative H&E images of the small intestine and quantitative analysis of intestine villus length, (g) representative images and quantification of PAS staining and (h) representative images and scoring of ZO-1 staining in the small intestine. Scale bar = 100 µm. ***, *p* < 0.001, **, *p* < 0.01, *, *p* < 0.05; ns, not significant.

The composition of gut microflora between EE and SE mice bearing PDAC was investigated using 16S rRNA sequencing of fecal samples collected at day 7 and day 21 post-tumor implantation (Figure 2(a)). As shown in Figure 2(b), at day 21 after tumor transplantation, the α-diversity of the gut microbiota was reduced by PDAC in SE group. Combined analysis of the fecal 16S rRNA sequencing results from day 7 and day 21 also showed that α-diversity was significantly higher in the EE group compared with the SE group (*p* < 0.05 for both Chao1 index and Shannon index, Figure 2(c)). Consistently, the Venn diagram showed there were more unique OTUs in EE mice than in SE mice (Figure 2(d)). Principal Coordinate Analysis (PCoA) revealed a clear distinction in gut microbiota between the EE and SE groups based on Weighted UniFrac metrics (Figure 2(e)).

The height of intestinal villi (*p* < 0.01, Figure 2(f)) and the number of the goblet cells (*p* < 0.001, Figure 2(g)) were significantly increased in EE mice. Additionally, the expression of intestinal epithelial marker ZO-1 (*p* < 0.01, Figure 2(h)) was remarkably upregulated in EE mice and the goblet-cell-specific mucin MUC2 expression had an increasing tendency (Figure S1a). To explore the gut barrier function of EE housing in tumor-bearing mice, we performed a FITC-dextran permeability assay. The passage of FITC-dextran across the intestinal barrier was elevated in SE tumor-bearing mice and this elevation disappeared in EE tumor-bearing mice (Figure S1B), which indicates part of restoration toward gut normobiosis by EE. These findings demonstrate that EE exerts a protective effect on the physical and chemical barriers of the intestinal mucosa.

### Eustress leads to the enrichment of *Lactobacillus* in mice with pancreatic cancer

**Figure 3.**
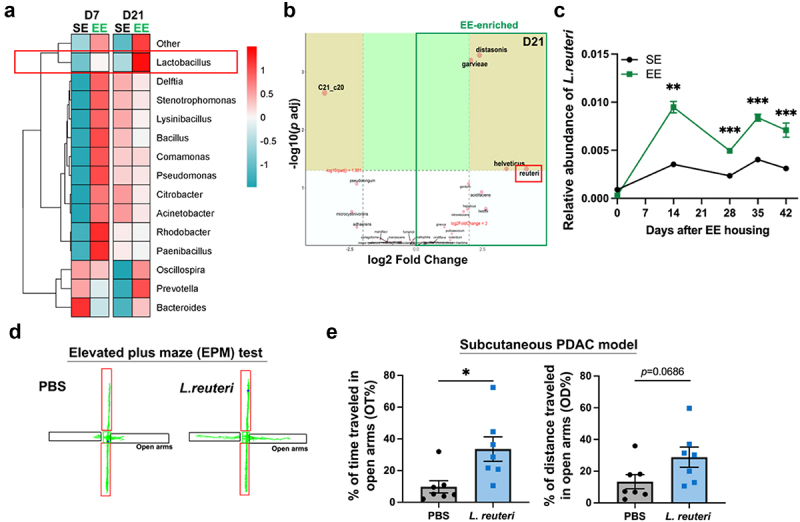
EE leads to the enrichment of *Lactobacillus* in mice with pancreatic cancer. (a) The heatmap displayed the relative abundance of the top 15 gut bacterial genera that contributed to the EE-induced tumor inhibition by abundance analysis. At the genus level, *Lactobacillus* was the only one consistently upregulated by EE at both day 7 and day 21. (b) The volcano plot showed the distinct taxa between EE and SE groups at day 21 by *DESeq2* analysis. At the species level, *L. reuteri* was the most upregulated species in the EE group. (c) qPCR quantification analysis confirmed a consistent increase in the fecal relative abundance of *L. reuteri* in the EE group. Compared with the PBS-treated control mice, the *L. reuteri-*treated mice exhibited reduced anxiety-like behaviors after 3 weeks of administration, as evidenced by (d) representative images and (e) the percentage of distance traveled in open arms and the percentage of time spent in open arms during the EPM test. ***, *p* < 0.001, **, *p* < 0.01, *, *p* < 0.05.

To determine which microbial populations significantly change with EE, we compared the gut bacterial abundance at the genus level between tumor-bearing EE and SE mice. Among the 15 most abundant gut bacterial genera, *Lactobacillus* was the only one consistently upregulated by EE at both day 7 and day 21 (Figure 3(a)). The *DESeq2* analysis revealed that at the species level, EE led to a 4.15-fold increase in the abundance of *L. reuteri* (FDR-adjusted *p* < 0.05, Figure 3(b)). qPCR results demonstrated that EE housing significantly increased the relative abundance of *L. reuteri* compared with SE housing (Figure 3(c)). These results suggest that *L. reuteri* is an inducible bacteria taxon in response to eustress.

Consistent with our previous reports,^15–18^ EE led to a significant tumor inhibition (Figure S2A-C), along with significantly lower levels of anxiety-like behaviors in the EPM test with obvious further distance and longer time traveled in open arms (*p* < 0.05, Figure S2D-E). Interestingly, the tumor-bearing mice treated with *L. reuteri* for three weeks also showed significantly decreased anxiety-like behaviors (the percent of time traveled in open arms, PBS group: 9.75 ± 3.88%, *L. reuteri* group: 33.56 ± 7.72%, *p* < 0.05, Figure 3(d–e)). These data indicate that *L. reuteri* is an EE-induced psychobiotic which may have positive feedback on mood.

### *Lactobacillus spp.* inhibit pancreatic cancer growth

**Figure 4.**
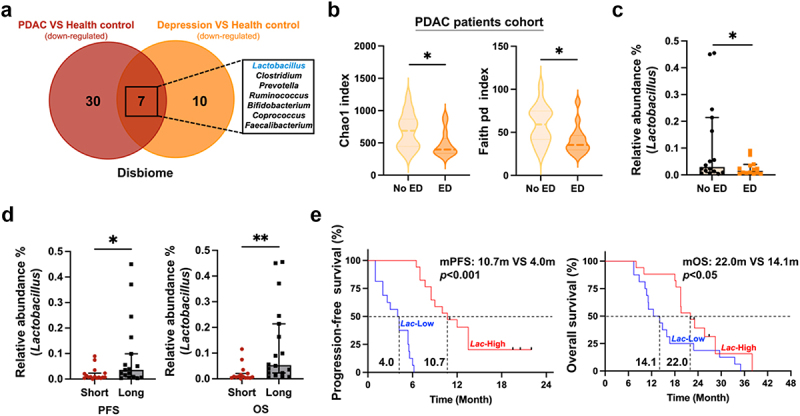
The abundance of *Lactobacillus* is associated with psychological stress and survival outcomes in patients with PDAC. (a) The venn diagram illustrated the shared genera associated with human PDAC and depression from the disbiome database. (b) Compared with the no emotional distress (no ED) group (*n* = 16), the α diversity in gut microbiome was lower in the emotional distress (ED) group (*n* = 14) in cohort 1. (c) Compared with the no ED group, the fecal relative abundance of *Lactobacillus* was lower in the ED group revealed by 16S rRNA sequencing in cohort 1. (d) Compared with the short survival group (*n* = 16), the fecal relative abundance of *Lactobacillus* was higher in the long survival group (*n* = 17) revealed by 16S rRNA sequencing in cohort 2. (e) Kaplan–Meier curve analysis of PFS (left) and OS (right) showed differences between patients with lower (*n* = 16) or higher (*n* = 17) fecal *Lactobacillus* relative abundance in cohort 2. *, *p*< 0.05, **, *p*< 0.01.

To explore the role of *Lactobacillus* in stress-related diseases and PDAC, we first examined the Disbiome public dataset (https://disbiome.ugent.be) for microbial taxa associated with depression and PDAC. We identified a total of 17 bacterial species significantly downregulated in patients with depressive disorders and 37 bacterial species significantly downregulated in PDAC patients (Figure 4(a)). The Venn diagram indicated that *Lactobacillus*, along with six other bacteria, such as *Clostridium*, *Prevotella*, *Ruminococcus*, *Bifidobacterium*, *Coprococcus* and *Faecalibacterium*, were present in the intersection of both groups, suggesting *Lactobacillu*s’ involvement in both diseases.

To further evaluate the clinical relevance of *Lactobacillus* in PDAC patients, we characterized the bacterial compositions in stool of 30 PDAC patients using 16S rRNA sequencing. Based on the Self-Rating Anxiety Scale (SAS) and Self-Rating Depression Scale (SDS), patients were categorized into two groups: those with emotional distress (ED, *n* = 14) and those without (no ED, *n* = 16). The richness of gut microbial species in the PDAC patients with emotional issues significantly decreased compared with those without emotional distress (*p* < 0.05, Figure 4(b)). PDAC patients with emotional issues demonstrated a lower abundance of *Lactobacillus* with borderline significance compared with those without emotional distress (median 0.03% vs. 0.01%, *p* < 0.05, Figure 4(c)). Meanwhile, the relative abundance of *Lactobacillus* was significantly associated with progression-free survival (PFS) and overall survival (OS, Figure 4(d)). According to the mean relative abundance of *Lactobacillus* in stool, PDAC patients were classified into the low *Lactobacillus* and high *Lactobacillus* groups. The median PFS was 4.0 months in the low *Lactobacillus* group, compared with 10.7 months in the high *Lactobacillus* group (*p* < 0.001, Figure 4(e), left). The median OS was 14.1 months in the low abundance of *Lactobacillus* group and 22.0 months in the high group (*p* < 0.05, Figure 4(e), right). These clinical observations supported the anti-tumor role of *Lactobacillus* in human PDAC.

**Figure 5.**
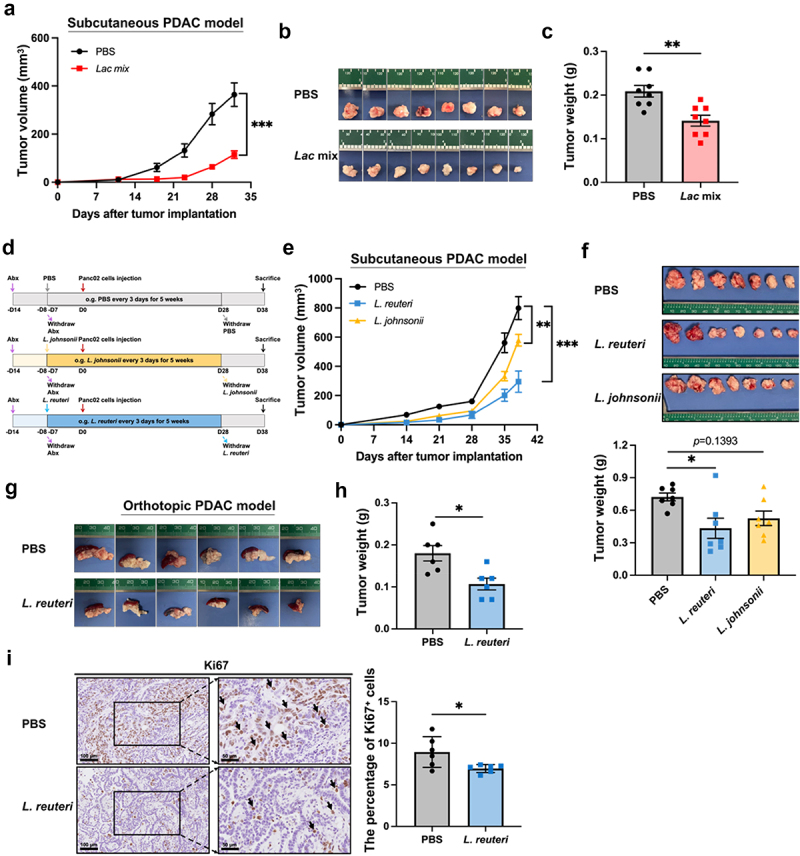
*Lactobacillus spp*. inhibit pancreatic tumor growth in mice. Abx-treated C57BL/6 mice received *Lac* mix administration containing *L. reuteri* and *L. johnsonii* and then Panc02 cell implantation. The *Lac* mix administration significantly restricted tumor growth, as supported by (a) tumor volume, (b) tumor images and (c) tumor weight in the subcutaneous PDAC model (*n* = 8/group). (d) Schematic diagram of *L. reuteri* or *L. johnsonii* administration in mice with pancreatic cancer. The administration of *L. reuteri* largely inhibited the Panc02 tumor growth, as evidenced by (e) tumor volume, (f) tumor images (top) and tumor weight (bottom) in the subcutaneous PDAC model (*n* = 7/group). The administration of *L. reuteri* inhibited orthotopic tumor growth, as shown by (g) tumor images and (h) tumor weight (*n* = 6/group). Fewer Ki67^+^ cancer cells in tumor tissue were observed in the *L. reuteri* group, as evidenced by (i) representative images and quantification of Ki67^+^ cells (*n* = 6/group). Scale bar (left) = 100 μm, scale bar (right) = 50 μm. ***, *p* < 0.001, **, *p* < 0.01, *, *p* < 0.05; ns, not significant.

To further confirm the anti-PDAC role of *Lactobacillus*, we gavaged PDAC-bearing mice with *Lactobacillus* mixture (*Lac* mix) containing two common species in the *Lactobacillus* genus, namely *L. reuteri* and *L. johnsonii*. Colonization with *Lac* mix remarkably inhibited Panc02 cells-derived tumor growth (Figure 5(a–b)). By the time of sacrifice at 32 days after tumor implantation, the tumor weight in the PBS group was 0.21 ± 0.01 g, whereas in the *Lac* mix group, it was only 0.14 ± 0.01 g (*p* < 0.01, Figure 5(c)). To identify which species exerts a better anti-tumor effect, we treated tumor-bearing mice separately with either *L. reuteri* or *L. johnsonii* (Figure 5(d)). As demonstrated in Figure 5(e,f) the administration of *L. reuteri* exerted a stronger inhibitory effect on tumor growth than *L. johnsonii*. By the end of the experiment at 38 days after tumor implantation, the tumor weights in the PBS, *L. reuteri*, and *L. johnsonii* groups were 0.72 ± 0.04 g, 0.43 ± 0.09 g, and 0.53 ± 0.07 g, respectively (PBS *vs L. reuteri* group: *p* < 0.05, PBS *vs. L. johnsonii* group: *p* > 0.05, Figure 5(f)). In addition to the subcutaneous tumor model, the anti-tumor efficacy of *L. reuteri* was also confirmed in the mice orthotopic model (PBS group: 0.18 ± 0.01 g, *L. reuteri* group: 0.10 ± 0.01 g, *p* < 0.05, Figure 5(g–h)). The IHC results showed a significantly lower percentage of Ki67^+^ cells in tumor sections of *L. reuteri*-treated mice than that in the control group (*p* < 0.05, Figure 5(i)). The colonization of *L. reuteri* showed a reduced trend in the frequency of liver metastases (Figure S3A). In both subcutaneous and orthotopic models of PDAC, the *L. reuteri* supplement did not affect the body weight of the mice (Figure S3B-C). Interestingly, the Firmicutes/Bacteroidetes ratio of the gut microbiome in the *L. reuteri* group was significantly higher than that in the PBS group (Figure S3D), revealing that *L. reuteri* administration contributes to the better maintenance of healthy gut homeostasis.^40^

### NK cells play an important role in the anti-tumor effect of *L. reuteri*

**Figure 6.**
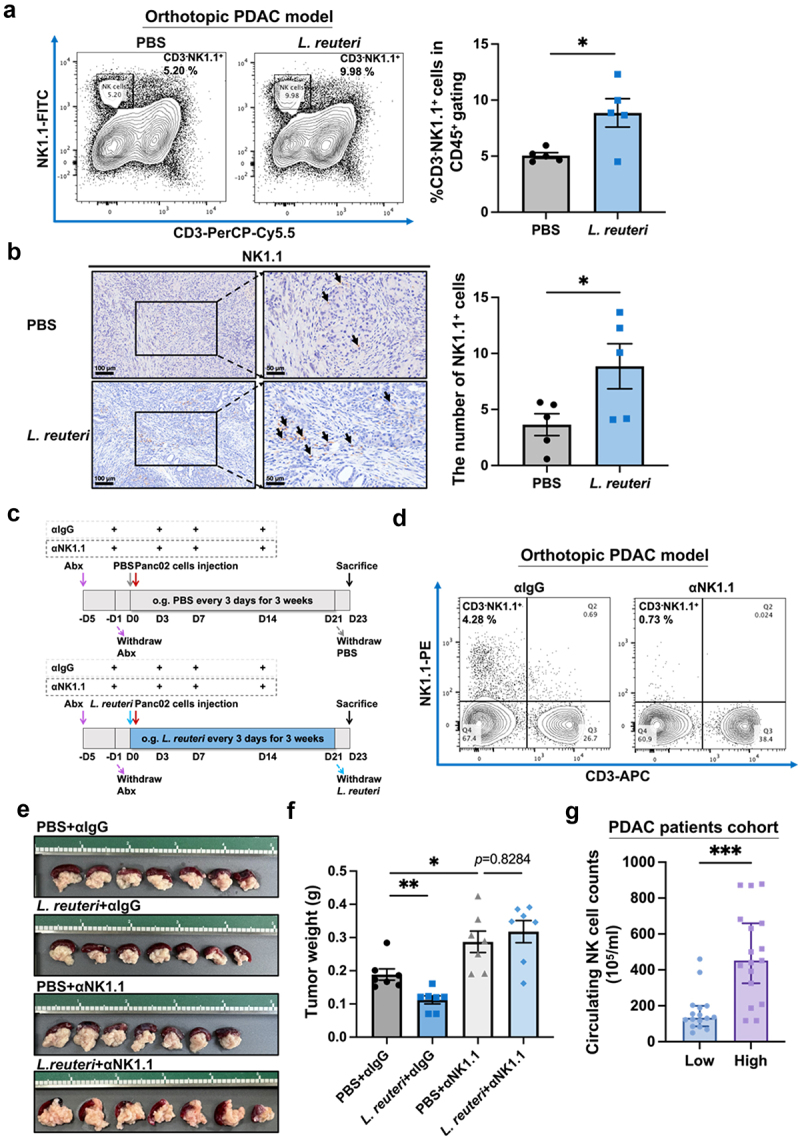
NK cells play an important role in the anti-tumor effect of *L. reuteri*. (a) Representative flow cytometry plots and quantification of NK cells (CD3^−^NK1.1^+^) suggested a significant upregulation of NK cells in the spleen of *L. reuteri*-treated mice compared with that of PBS-treated control mice (*n* = 5/group). (b) IHC staining confirmed a significant increase in NK1.1^+^ cells in the tumor tissues of *L. reuteri*-treated mice. Scale bar (left) = 100 μm, scale bar (right) = 50 μm. (c) Schematic diagram of NK cell depletion in the orthotopic PDAC model (*n* = 7/group). The administration of anti-NK1.1 monoclonal antibody negated the tumor-inhibitory effect of *L. reuteri*, as evidenced by (d) representative flow cytometry plots of NK cells (CD3^−^NK1.1^+^) in the spleen which confirmed the success of NK cell depletion, (e) tumor images and (f) tumor weight. (g) In cohort 2, circulating NK cell counts were increased in PDAC patients with higher fecal *Lactobacillus* relative abundance (*n* = 17) compared with those with lower relative abundance (*n* = 16). ***, *p* < 0.001, **, *p* < 0.01, *, *p* < 0.05; ns, not significant.

To answer whether the immune system was involved in *L. reuteri*-induced anti-tumor activity, we examined the percentage of NK, NKT, B and CD8^+^ T cells in the spleen and orthotopic tumor tissues of the *L. reuteri*-treated mice. Flow cytometry results revealed a significant increase in the percentage of NK cells (CD3^−^ NK1.1^+^) in the spleen following *L. reuteri* treatment (*p* < 0.05, Figure 6(a)). The IHC staining results demonstrated a significant increase in the proportion of NK cells infiltrating the TME in the *L. reuteri* group (*p* < 0.05, Figure 6(b)). Aside from NK cells, there were no significant changes in the percentage of other tested lymphocytes, namely B cells (CD19^+^), CD8^+^ T cells (CD3^+^CD8^+^) and NKT cells (CD3^+^NK1.1^+^), in the spleen of *L. reuteri-*treated group compared with that of the control group (Figure S4A-D).

To further validate the role of NK cells in the anti-tumor effect of *L. reuteri*, we depleted NK cells *in vivo* by injecting an anti-NK1.1 antibody (Figure 6(c)). The FACS analysis indicated the successful depletion of NK cells *in vivo* (Figure 6(d)). Notably, the depletion of NK cells almost completely abolished the tumor suppressive effect of *L. reuteri* (PBS+αIgG *vs*. PBS+αNK1.1 group: *p* < 0.05, PBS+αNK1.1 *vs. L. reuteri*+αNK1.1 group: *p* > 0.05, Figure 6(e–f)), indicating NK immunity mediates the anti-tumor effect of *L. reuteri*.

Clinically, we analyzed the relative abundance of *Lactobacillus* in stool and the absolute number of circulating NK cells in peripheral blood (Figure 6(g)). The PDAC patients were divided into two groups based on the relative abundance of *Lactobacillus* in stool. The median value of peripheral circulating NK cell counts in the low *Lactobacillus* group was 1.33 × 10^7^ cells/mL, while that in the high *Lactobacillus* group was 4.52 × 10^7^ cells/mL (*p* < 0.001, Figure 6(g)). These results suggest that the high relative abundance of *Lactobacillus* correlates with increased peripheral circulating NK cells in PDAC patients.

## Discussion

**Figure 7.**
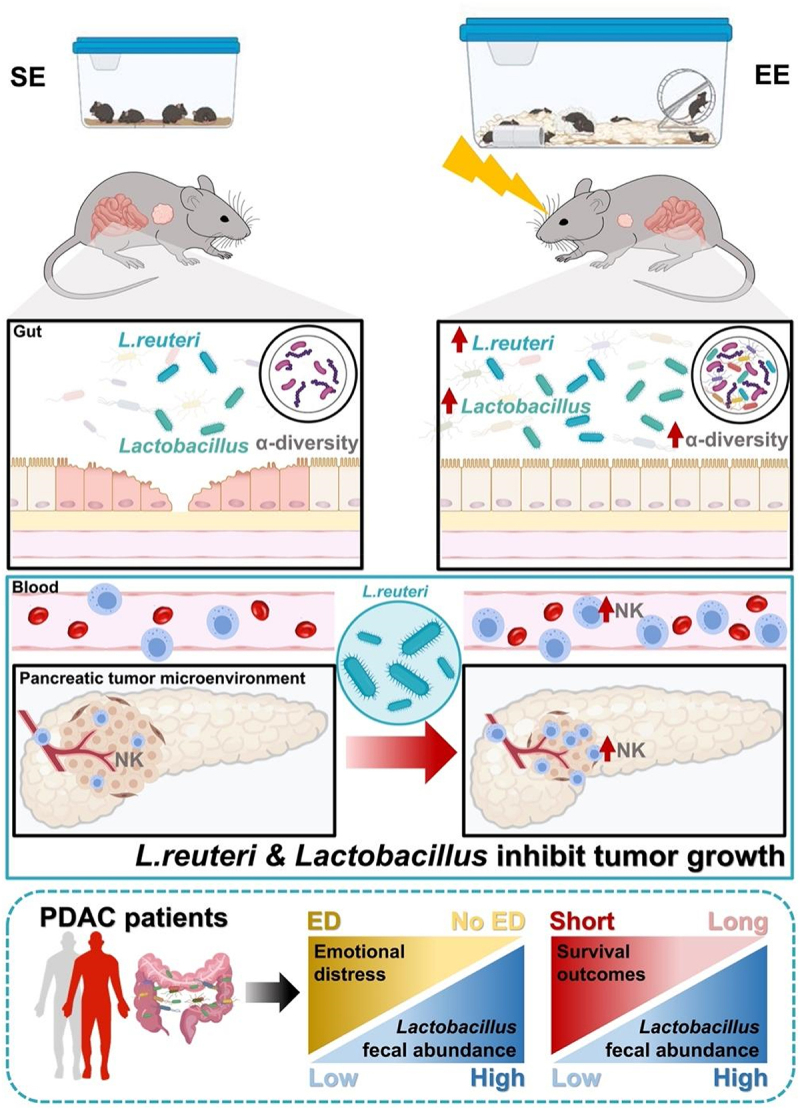
Mechanism diagram of Eustress-derived *Lactobacillus spp*. attenuate tumor growth inhibition. Gut microbiota signals play a crucial role in the tumor-suppressive effects of EE. Notably, EE significantly enriches the abundance of *Lactobacillus spp*., particularly the *L. reuteri*, which induces a robust anti-PDAC phenotype in mice via NK cell-mediated immunity. Clinically, PDAC patients with lower levels of *Lactobacillus* are more susceptible to ED and poor survival outcomes.

Mounting evidence has consistently supported that eustress induced by EE has a potent inhibitory effect on cancer. However, the mechanisms by which the brain regulates peripheral tumor growth remain largely unknown. Cao *et al*. proposed a brain-adipocyte axis to explain the mechanism underlying the cancer remission and inhibition caused by EE.^13^ Our previous studies have demonstrated that EE suppresses pancreatic cancer by modulating NK cells via the sympathetic nervous system.^15^ In the current study, we revealed a novel mechanism by which EE inhibits PDAC. We found that EE mitigates tumor-induced gut dysbiosis and barrier damage. Through microbiota depletion and FMT experiments, we confirmed that the gut microbiota plays a critical role in the tumor-suppressive effects of EE. Moreover, comparative analyses of the fecal microbiota from tumor-bearing mice housed in EE versus SE, along with the analysis of clinical data, allowed us to identify *L. reuteri* as one of the most dominant flora in the gut, which is sensitive to EE stimulation and simultaneously processes tumor-suppressing properties (Figure 7). These findings open up a new direction for the adjuvant therapy of PDAC.

Housing in EE condition is a classic eustress model that provides animals with social, sensory and motor stimuli. EE exerts beneficial effects on various disorders related to the nervous system including depression, anxiety, neurodegenerative conditions and stroke,^41^ as well as non-neuropsychiatric diseases such as nonalcoholic steatohepatitis,^42^ myocardial infarction^43^ and cancers.^44^ Emerging evidence suggests that EE exposure can modify gut microbiome composition in healthy mice^45^ and disease models.^42,46,47^ In the context of cancer, the impact of EE on the gut-brain-microbiota axis has been investigated, with a primary focus on colorectal cancer. In a *Tcf4*^Het/+^*Apc*^Min/+^-mediated model of colon tumorigenesis, EE drastically increased the relative abundance of *Proteobacteria* and *Sutterella* within the colon.^24^ In the rat model of 1,2-dimethylhydrazine (DMH)-induced colorectal cancer, EE was found to have beneficial effect on the intestinal mucosal barrier.^48,49^ Aside from these descriptive reports, little is known about the genuine contribution of microbiota changes to the EE-induced tumor suppression phenotype, particularly for extra-intestinal tumors. Our study thus investigated the impact of EE on gut microbiota composition using syngeneic mouse models of pancreatic cancer. We observed an increase in the α-diversity of gut bacterial community under EE exposure. More importantly, we demonstrated for the first time that FMT with feces from EE mice can significantly reduce tumor burden in recipient SE mice. To our knowledge, donor feces with such eustressful “anti-tumor” potential have not been previously reported. It is speculated that if it is hard to replicate the eustressful conditions in PDAC patients, transplanting stool from healthy donors who live positive lives may induce a similar anti-tumor effect in the recipients.

Previous studies in humans and laboratory rodents reported that the abundance of *Lactobacillus* in the gut was reduced after stress.^50^ In our study, *Lactobacillus* was upregulated in response to EE stimulation on both day 7 and day 21 post-tumor implantation. This finding provided additional evidence from the perspective of eustress to support the notion that *Lactobacillus* is a mood-related bacterium. Supplementation with *L. reuteri* reduced anxiety levels in tumor-bearing mice, further indicating that *L. reuteri* plays a bidirectional role in brain-gut communication. To date, how eustress influences intestinal *Lactobacillus spp*. has not been reported. However, a recent study by Araujo’s group found that restraint-induced distress significantly decreased the abundance of gut *Lactobacillus spp*. by inhibiting mucin secretion via a central amygdala-vagal-duodenal glandular circuit.^51^ Notably, we observed a substantial increase in the intestinal mucin protein MUC2 in EE-treated mice (Figure S1A). Based on these findings, it is reasonable to hypothesize that eustress may similarly leverage this circuit to upregulate *Lactobacillus spp*. This hypothesis will be tested in our future studies.

*L. reuteri* is a Gram-positive, rod-shaped lactic acid bacterium. As a gut symbiont, *L. reuteri* performs multiple functions including regulating immune responses, modulating gut microbiota balance, boosting beneficial metabolites and maintaining barrier function. Emerging evidence suggests that *L. reuteri* can inhibit the growth of certain types of cancer such as melanoma^52,53^ and colorectal cancer.^54,55^ Regarding pancreatic cancer, *L. reuteri* administration combined with *Lactobacillus paracasei* or *Lactobacillus casei* (*L. casei*) has been reported to repress pancreatic cancer growth in nude mice xenografted with PDAC cells^56^ and attenuate the cancer progression promoted by *Porphyromonas gingivalis* in *K-ras*^*G12D*^ transgenic mice.^57^ The tumor suppressive effect of *L. reuteri* on its own on PDAC has not been explored previously. Our study demonstrated that *L. reuteri* can elicit a 40% tumor inhibition rate in the subcutaneous PDAC mice model, which is comparable to that with *L. reuteri* plus *L. casei* treatment.^57^ In addition, in the clinical set, the abundance of *L. reuteri* was found to be closely correlated with PFS, supporting that the supplementation of *L. reuteri* might confer tumor resistance in patients with PDAC, particularly those who are experiencing emotional challenges.

The percentage of NK cells was increased after intragastric administration of *L. reuteri*, whereas B cells and CD8^+^ T cells did not show significant changes in tumor-bearing mice under EE, suggesting that innate immunity plays a part in the anti-tumor effect induced by *L. reuteri*. NK cell depletion experiment further supported this speculation. Previously, studies regarding the anti-tumor effects of *L. reuteri* have focused on metabolites.^52,54^ This study proposed a novel mechanism through which psychobiotic *L. reuteri* exerts its anti-tumor function via NK cells. Although we have demonstrated the important role of *L. reuteri* in NK functionality against PDAC, the molecular mechanisms of the interplay between *L. reuteri* and NK immunity in PDAC remain to be elucidated in future studies. Currently, direct evidence explaining this link is lacking. A recent study found that transplantation of *L. reuteri* increases serum acetate levels.^58^ Interestingly, another study reported that acetate supplementation enhances T cell and NK cell functions, thereby potentiating anti-tumor immunity in breast cancer.^59^ These reports lead us to speculate that acetate, or other short-chain fatty acids, may also mediate the enhancement of NK cell immunity under *L. reuteri* treatment in pancreatic cancer. Besides *L. reuteri*, several other bacteria species within the genus *Lactobacillus*, such as *Lactobacillus rhamnosus* ^60^ and *Lactobacillus delbrueckii ssp. bulgaricus*,^61^ are also capable of activating NK cells. They achieve this through bacteria-specific exopolysaccharides (EPS), which stimulate NK cell proliferation and interferon production. Therefore, the identification of polysaccharides with NK cell activation properties in *L. reuteri* will aid in elucidating its molecular mechanisms.

Our study has several limitations. First, we do not investigate the details of how eustress upregulates gut *Lactobacillus spp*. and how *Lactobacillus spp*. enhance NK cell immunity. These important mechanisms warrant in-depth investigation in the future. Second, the sample size of the clinical study is relatively small. Further large-scale, multi-center studies are needed to confirm the relationships between ED, gut *Lactobacillus*, and pancreatic cancer outcomes. Third, the use of second-generation sequencing technology limits the efficiency of identifying differential bacteria between EE and SE mice. Future research utilizing metagenomics or third-generation sequencing approaches will be valuable for identifying additional differential bacteria at the species level.

In summary, our study introduces a novel concept: gut microbiota signals contribute to the tumor-suppressive effects of eustress. *L. reuteri*, a psychobiotic influenced by EE, can induce a strong anti-PDAC phenotype in mice via NK immunity. Given that patients with PDAC are accompanied by gut dysbiosis, and those lacking *Lactobacillus* have poor outcomes, it is reasonable to speculate that oral administration of *L. reuteri* would be an encouraging therapeutic strategy in the treatment of pancreatic cancer.

## Supplementary Material

Supplemental Material

## Data Availability

The original contributions presented in the study are included in the article/supplementary material, further inquiries can be directed to the corresponding authors. 16S rRNA gene sequencing data are available in the China National Center for Bioinformation (https://ngdc.cncb.ac.cn) under accession number PRJCA031012.

## References

[1] Siegel RL, Giaquinto AN, Jemal A. Cancer statistics, 2024. CA Cancer J Clin. 2024;74(1):12–18. doi:10.3322/caac.21820. 38230766

[2] Wu Y, Zhou L, Zhang X, Yang X, Niedermann G, Xue J. Psychological distress and eustress in cancer and cancer treatment: advances and perspectives. Sci Adv. 2022;8(47):eabq7982. doi:10.1126/sciadv.abq7982. 36417542 PMC9683699

[3] Eckerling A, Ricon-Becker I, Sorski L, Sandbank E, Ben-Eliyahu S. Stress and cancer: mechanisms, significance and future directions. Nat Rev Cancer. 2021;21(12):767–785. doi:10.1038/s41568-021-00395-5. 34508247

[4] Fraterman I, Reijers ILM, Dimitriadis P, Broeks A, Gonzalez M, Menzies AMM, Lopez-Yurda M, Kapiteijn E, van der Veldt AAM, Suijkerbuijk KPM, et al. Association between pretreatment emotional distress and neoadjuvant immune checkpoint blockade response in melanoma. Nat Med. 2023;29(12):3090–3099. doi:10.1038/s41591-023-02631-x. 37957378

[5] Zeng Y, Hu CH, Li YZ, Zhou JS, Wang SX, Liu MD, Qiu ZH, Deng C, Ma F, Xia CF, et al. Association between pretreatment emotional distress and immune checkpoint inhibitor response in non-small-cell lung cancer. Nat Med. 2024;30(6):1680–1688. doi:10.1038/s41591-024-02929-4. 38740994 PMC11186781

[6] Peduzzi G, Felici A, Pellungrini R, Giorgolo F, Farinella R, Gentiluomo M, Spinelli A, Capurso G, Monreale A, Canzian F, et al. Analysis of exposome and genetic variability suggests stress as a major contributor for development of pancreatic ductal adenocarcinoma. Dig Liver Dis. 2024;56(6):1054–1063. doi:10.1016/j.dld.2023.10.015. 37985251

[7] Batty GD, Russ TC, Stamatakis E, Kivimäki M. Psychological distress in relation to site specific cancer mortality: pooling of unpublished data from 16 prospective cohort studies. Bmj. 2017;356:j108. doi:10.1136/bmj.j108. 28122812 PMC5266623

[8] Carney CP, Jones L, Woolson RF, Noyes R Jr., Doebbeling BN. Relationship between depression and pancreatic cancer in the general population. Psychosom Med. 2003;65(5):884–888. doi:10.1097/01.psy.0000088588.23348.d5. 14508036

[9] Yang H, Xia L, Chen J, Zhang S, Martin V, Li Q, Lin S, Chen J, Calmette J, Lu M, et al. Stress–glucocorticoid–TSC22D3 axis compromises therapy-induced antitumor immunity. Nat Med. 2019;25(9):1428–1441. doi:10.1038/s41591-019-0566-4. 31501614

[10] Thaker PH, Han LY, Kamat AA, Arevalo JM, Takahashi R, Lu C, Jennings NB, Armaiz-Pena G, Bankson JA, Ravoori M, et al. Chronic stress promotes tumor growth and angiogenesis in a mouse model of ovarian carcinoma. Nat Med. 2006;12(8):939–944. doi:10.1038/nm1447. 16862152

[11] Le CP, Nowell CJ, Kim-Fuchs C, Botteri E, Hiller JG, Ismail H, Pimentel MA, Chai MG, Karnezis T, Rotmensz N, et al. Chronic stress in mice remodels lymph vasculature to promote tumour cell dissemination. Nat Commun. 2016;7(1):10634. doi:10.1038/ncomms10634. 26925549 PMC4773495

[12] Ma Y, Kroemer G. The cancer-immune dialogue in the context of stress. Nat Rev Immunol. 2024;24(4):264–281. doi:10.1038/s41577-023-00949-8. 37833492

[13] Cao L, Liu X, Lin EJ, Wang C, Choi EY, Riban V, Lin B, During MJ. Environmental and genetic activation of a brain-adipocyte BDNF/Leptin axis causes cancer remission and inhibition. Cell. 2010;142(1):52–64. doi:10.1016/j.cell.2010.05.029. 20603014 PMC3784009

[14] Cao L, During MJ. What is the brain-cancer connection? Annu Rev Neurosci. 2012;35(1):331–345. doi:10.1146/annurev-neuro-062111-150546. 22462541

[15] Song Y, Gan Y, Wang Q, Meng Z, Li G, Shen Y, Wu Y, Li P, Yao M, Gu J, et al. Enriching the housing environment for mice enhances their NK cell antitumor immunity via sympathetic nerve–dependent regulation of NKG2D and CCR5. Cancer Res. 2017;77(7):1611–1622. doi:10.1158/0008-5472.can-16-2143. 28082402

[16] Li G, Gan Y, Fan Y, Wu Y, Lin H, Song Y, Cai X, Yu X, Pan W, Yao M, et al. Enriched environment inhibits mouse pancreatic cancer growth and down-regulates the expression of mitochondria-related genes in cancer cells. Sci Rep. 2015;5(1):7856. doi:10.1038/srep07856. 25598223 PMC4297951

[17] Wu Y, Gan Y, Yuan H, Wang Q, Fan Y, Li G, Zhang J, Yao M, Gu J, Tu H. Enriched environment housing enhances the sensitivity of mouse pancreatic cancer to chemotherapeutic agents. Biochem Biophys Res Commun. 2016;473(2):593–599. doi:10.1016/j.bbrc.2016.03.128. 27033603

[18] Meng Z, Liu T, Song Y, Wang Q, Xu D, Jiang J, Li M, Qiao J, Luo X, Gu J, et al. Exposure to an enriched environment promotes the terminal maturation and proliferation of natural killer cells in mice. Brain Behav Immun. 2019;77:150–160. doi:10.1016/j.bbi.2018.12.017. 30590110

[19] Bergin SM, Xiao R, Huang W, Judd CRT, Liu X, Mansour AG, Queen N, Widstrom KJ, Caligiuri MA, Cao L. Environmental activation of a hypothalamic bdnf-adipocyte IL-15 axis regulates adipose-natural killer cells. Brain Behav Immun. 2021;95:477–488. doi:10.1016/j.bbi.2021.05.005. 33989745 PMC8493653

[20] Xiao R, Bergin SM, Huang W, Slater AM, Liu X, Judd RT, Lin ED, Widstrom KJ, Scoville SD, Yu J, et al. Environmental and genetic activation of hypothalamic BDNF modulates T-cell immunity to exert an anticancer phenotype. Cancer Immunol Res. 2016;4(6):488–497. doi:10.1158/2326-6066.cir-15-0297. 27045020 PMC4891265

[21] Xiao R, Bergin SM, Huang W, Mansour AG, Liu X, Judd RT, Widstrom KJ, Queen NJ, Wilkins RK, Siu JJ, et al. Enriched environment regulates thymocyte development and alleviates experimental autoimmune encephalomyelitis in mice. Brain Behav Immun. 2019;75:137–148. doi:10.1016/j.bbi.2018.09.028. 30287389 PMC6279528

[22] Gurfein BT, Hasdemir B, Milush JM, Touma C, Palme R, Nixon DF, Darcel N, Hecht FM, Bhargava A, Akiyama T. Enriched environment and stress exposure influence splenic B lymphocyte composition. PLOS ONE. 2017;12(7):e0180771. doi:10.1371/journal.pone.0180771. 28704473 PMC5507530

[23] Wang Q, Li M, Gan Y, Jiang S, Qiao J, Zhang W, Fan Y, Shen Y, Song Y, Meng Z, et al. Mitochondrial protein UQCRC1 is oncogenic and a potential therapeutic target for pancreatic cancer. Theranostics. 2020;10(5):2141–2157. doi:10.7150/thno.38704. 32089737 PMC7019160

[24] Bice BD, Stephens MR, Georges SJ, Venancio AR, Bermant PC, Warncke AV, Affolter KE, Hidalgo JR, Angus-Hill ML. Environmental enrichment induces pericyte and IgA-dependent wound repair and lifespan extension in a colon tumor model. Cell Rep. 2017;19(4):760–773. doi:10.1016/j.celrep.2017.04.006. 28445727 PMC5474344

[25] Hanahan D. Hallmarks of cancer: new dimensions. Cancer Discov. 2022;12(1):31–46. doi:10.1158/2159-8290.cd-21-1059. 35022204

[26] Ren Z, Jiang J, Xie H, Li A, Lu H, Xu S, Zhou L, Zhang H, Cui G, Chen X, et al. Gut microbial profile analysis by MiSeq sequencing of pancreatic carcinoma patients in China. Oncotarget. 2017;8(56):95176–95191. doi:10.18632/oncotarget.18820. 29221120 PMC5707014

[27] Kartal E, Schmidt TSB, Molina-Montes E, Rodríguez-Perales S, Wirbel J, Maistrenko OM, Akanni WA, Alashkar Alhamwe B, Alves RJ, Carrato A, et al. A faecal microbiota signature with high specificity for pancreatic cancer. Gut. 2022;71(7):1359–1372. doi:10.1136/gutjnl-2021-324755. 35260444 PMC9185815

[28] Zhou W, Zhang D, Li Z, Jiang H, Li J, Ren R, Gao X, Li J, Wang X, Wang W, et al. The fecal microbiota of patients with pancreatic ductal adenocarcinoma and autoimmune pancreatitis characterized by metagenomic sequencing. J Transl Med. 2021;19(1):215. doi:10.1186/s12967-021-02882-7. 34006295 PMC8130326

[29] Kharofa J, Haslam D, Wilkinson R, Weiss A, Patel S, Wang K, Esslinger H, Olowokure O, Sohal D, Wilson G, et al. Analysis of the fecal metagenome in long-term survivors of pancreas cancer. Cancer. 2023;129(13):1986–1994. doi:10.1002/cncr.34748. 36943918

[30] Riquelme E, Zhang Y, Zhang L, Montiel M, Zoltan M, Dong W, Quesada P, Sahin I, Chandra V, San Lucas A, et al. Tumor microbiome diversity and composition influence pancreatic cancer outcomes. Cell. 2019;178(4):795–806.e12. doi:10.1016/j.cell.2019.07.008. 31398337 PMC7288240

[31] Abe S, Masuda A, Matsumoto T, Inoue J, Toyama H, Sakai A, Kobayashi T, Tanaka T, Tsujimae M, Yamakawa K, et al. Impact of intratumoral microbiome on tumor immunity and prognosis in human pancreatic ductal adenocarcinoma. J Gastroenterol. 2024;59(3):250–262. doi:10.1007/s00535-023-02069-5. 38242997 PMC10904450

[32] Zhang Z, Wang T, Xu M, Zhang Z, Wang H, Xue J, Wang W. Deciphering the pancreatic cancer microbiome in Mainland China: impact of Exiguobacterium/Bacillus ratio on tumor progression and prognostic significance. Pharmacol Res. 2024;204:107197. doi:10.1016/j.phrs.2024.107197. 38692467

[33] Pushalkar S, Hundeyin M, Daley D, Zambirinis CP, Kurz E, Mishra A, Mohan N, Aykut B, Usyk M, Torres LE, et al. The pancreatic cancer microbiome promotes oncogenesis by induction of innate and adaptive immune suppression. Cancer Discov. 2018;8(4):403–416. doi:10.1158/2159-8290.cd-17-1134. 29567829 PMC6225783

[34] Margolis KG, Cryan JF, Mayer EA. The microbiota-gut-brain axis: from motility to mood. Gastroenterology. 2021;160(5):1486–1501. doi:10.1053/j.gastro.2020.10.066. 33493503 PMC8634751

[35] Morais LH, Schreiber H, Mazmanian SK. The gut microbiota-brain axis in behaviour and brain disorders. Nat Rev Microbiol. 2021;19(4):241–255. doi:10.1038/s41579-020-00460-0. 33093662

[36] Kim H, Samuel S, Totenhagen JW, Warren M, Sellers JC, Buchsbaum DJ. Dynamic contrast enhanced magnetic resonance imaging of an orthotopic pancreatic cancer mouse model. J Vis Exp. 2015;2015(98). doi:10.3791/52641. PMC4541579 25938718

[37] Dai M, Lui RN, Lau LHS. The role of gut microbiome and fecal microbiota transplantation in liver cancer and related complications: mechanisms and therapeutic potentials. Hepatoma Res. 2023;9:39. doi:10.20517/2394-5079.2023.33.

[38] Komada M, Takao K, Miyakawa T. Elevated plus maze for mice. J Vis Exp. 2008;2008(22). doi:10.3791/1088. PMC2762911 19229173

[39] Wang R, Hasnain SZ. Analyzing the properties of murine intestinal mucins by electrophoresis and histology. Bio Protoc. 2017;7(14):e2394. doi:10.21769/BioProtoc.2394. PMC8413512 34541128

[40] Ma Z, Zuo T, Frey N, Rangrez AY. A systematic framework for understanding the microbiome in human health and disease: from basic principles to clinical translation. Signal Transduct Target Ther. 2024;9(1):237. doi:10.1038/s41392-024-01946-6. 39307902 PMC11418828

[41] Kempermann G. Environmental enrichment, new neurons and the neurobiology of individuality. Nat Rev Neurosci. 2019;20(4):235–245. doi:10.1038/s41583-019-0120-x. 30723309

[42] Higarza SG, Arboleya S, Arias JL, Gueimonde M, Arias N. Akkermansia muciniphila and environmental enrichment reverse cognitive impairment associated with high-fat high-cholesterol consumption in rats. Gut Microbes. 2021;13(1):1–20. doi:10.1080/19490976.2021.1880240. PMC7946069 33678110

[43] Bai PY, Chen SQ, Jia DL, Pan LH, Liu CB, Liu J, Luo W, Yang Y, Sun MY, Wan NF, et al. Environmental eustress improves postinfarction cardiac repair via enhancing cardiac macrophage survival. Sci Adv. 2022;8(17):eabm3436. doi:10.1126/sciadv.abm3436. 35476440 PMC9045726

[44] Liu C, Yang Y, Chen C, Li L, Li J, Wang X, Chu Q, Qiu L, Ba Q, Li X, et al. Environmental eustress modulates β-ARs/CCL2 axis to induce anti-tumor immunity and sensitize immunotherapy against liver cancer in mice. Nat Commun. 2021;12(1):5725. doi:10.1038/s41467-021-25967-9. 34593796 PMC8484272

[45] Marrocco F, Delli Carpini M, Garofalo S, Giampaoli O, De Felice E, Di Castro MA, Maggi L, Scavizzi F, Raspa M, Marini F, et al. Short-chain fatty acids promote the effect of environmental signals on the gut microbiome and metabolome in mice. Commun Biol. 2022;5(1):517. doi:10.1038/s42003-022-03468-9. 35641653 PMC9156677

[46] Singh Y, El-Hadidi M, Admard J, Wassouf Z, Schulze-Hentrich JM, Kohlhofer U, Quintanilla-Martinez L, Huson D, Riess O, Casadei N. Enriched environmental conditions modify the gut microbiome composition and fecal markers of inflammation in Parkinson’s disease. Front Neurosci. 2019;13:1032. doi:10.3389/fnins.2019.01032. 31749671 PMC6842954

[47] Lupori L, Cornuti S, Mazziotti R, Borghi E, Ottaviano E, Cas MD, Sagona G, Pizzorusso T, Tognini P. The gut microbiota of environmentally enriched mice regulates visual cortical plasticity. Cell Rep. 2022;38(2):110212. doi:10.1016/j.celrep.2021.110212. 35021093

[48] Dun L, Mei-Jing C, Si-Ting H, Xin-Yuan Y, Yu-Xuan W. Effects of different environmental intervention durations on the intestinal mucosal barrier and the brain-gut axis in rats with colorectal cancer. Sci Rep. 2022;12(1):20442. doi:10.1038/s41598-022-24861-8. 36443338 PMC9705392

[49] Liu D, Jiang XY, Zhou LS. Enriched environment on the intestinal mucosal barrier and brain-gut axis in rats with colorectal cancer. Exp Biol Med (Maywood). 2018;243(15–16):1185–1198. doi:10.1177/1535370218815437. 30486675 PMC6384444

[50] Galley JD, Bailey MT. Impact of stressor exposure on the interplay between commensal microbiota and host inflammation. Gut Microbes. 2014;5(3):390–396. doi:10.4161/gmic.28683. 24690880 PMC4153778

[51] Chang H, Perkins MH, Novaes LS, Qian F, Zhang T, Neckel PH, Scherer S, Ley RE, Han W, de Araujo IE. Stress-sensitive neural circuits change the gut microbiome via duodenal glands. Cell. 2024;187(19):5393–5412.e5330. doi:10.1016/j.cell.2024.07.019. 39121857 PMC11425084

[52] Bender MJ, McPherson AC, Phelps CM, Pandey SP, Laughlin CR, Shapira JH, Medina Sanchez L, Rana M, Richie TG, Mims TS, et al. Dietary tryptophan metabolite released by intratumoral Lactobacillus reuteri facilitates immune checkpoint inhibitor treatment. Cell. 2023;186(9):1846–1862.e26. doi:10.1016/j.cell.2023.03.011. 37028428 PMC10148916

[53] Luo M, Hu M, Feng X, XiaoLi W, Dong D, Wang W. Preventive effect of Lactobacillus reuteri on melanoma. Biomed Pharmacother. 2020;126:109929. doi:10.1016/j.biopha.2020.109929. 32126498

[54] Bell HN, Rebernick RJ, Goyert J, Singhal R, Kuljanin M, Kerk SA, Huang W, Das NK, Andren A, Solanki S, et al. Reuterin in the healthy gut microbiome suppresses colorectal cancer growth through altering redox balance. Cancer Cell. 2022;40(2):185–200.e6. doi:10.1016/j.ccell.2021.12.001. 34951957 PMC8847337

[55] Li N, Niu L, Liu Y, Wang Y, Su X, Xu C, Sun Z, Guo H, Gong J, Shen S. Taking SCFAs produced by Lactobacillus reuteri orally reshapes gut microbiota and elicits antitumor responses. J Nanobiotechnol. 2024;22(1):241. doi:10.1186/s12951-024-02506-4. PMC11089779 38735933

[56] Zhu Z, Yi B, Tang Z, Chen X, Li M, Xu T, Zhao Z, Tang C. Lactobacillus casei combined with Lactobacillus reuteri alleviate pancreatic cancer by inhibiting TLR4 to promote macrophage M1 polarization and regulate gut microbial homeostasis. BMC Cancer. 2023;23(1):1044. doi:10.1186/s12885-023-11557-z. 37904102 PMC10614400

[57] Chen SM, Hsu LJ, Lee HL, Lin CP, Huang SW, Lai CJ, Lin CW, Chen WT, Chen YJ, Lin YC, et al. Lactobacillus attenuate the progression of pancreatic cancer promoted by porphyromonas gingivalis in K-ras^G12D^ transgenic mice. Cancers (Basel). 2020;12(12):3522. doi:10.3390/cancers12123522. 33255941 PMC7760978

[58] Hu C, Xu B, Wang X, Wan WH, Lu J, Kong D, Jin Y, You W, Sun H, Mu X, et al. Gut microbiota–derived short-chain fatty acids regulate group 3 innate lymphoid cells in HCC. Hepatology. 2023;77(1):48–64. doi:10.1002/hep.32449. 35262957 PMC9970019

[59] Miller KD, O’Connor S, Pniewski KA, Kannan T, Acosta R, Mirji G, Papp S, Hulse M, Mukha D, Hlavaty SI, et al. Acetate acts as a metabolic immunomodulator by bolstering T-cell effector function and potentiating antitumor immunity in breast cancer. Nat Cancer. 2023;4(10):1491–1507. doi:10.1038/s43018-023-00636-6. 37723305 PMC10615731

[60] Le Noci V, Guglielmetti S, Arioli S, Camisaschi C, Bianchi F, Sommariva M, Storti C, Triulzi T, Castelli C, Balsari A, et al. Modulation of pulmonary microbiota by antibiotic or probiotic aerosol therapy: a strategy to promote immunosurveillance against lung metastases. Cell Rep. 2018;24(13):3528–3538. doi:10.1016/j.celrep.2018.08.090. 30257213

[61] Makino S, Sato A, Goto A, Nakamura M, Ogawa M, Chiba Y, Hemmi J, Kano H, Takeda K, Okumura K, et al. Enhanced natural killer cell activation by exopolysaccharides derived from yogurt fermented with Lactobacillus delbrueckii ssp. bulgaricus OLL1073R-1. J Dairy Sci. 2016;99(2):915–923. doi:10.3168/jds.2015-10376. 26686726

