# Supplementary material for: Upregulation of Lactobacillus spp. in gut microbiota as a novel mechanism for environmental eustress-induced anti-pancreatic cancer effects
Source: Gut Microbes. 2025 Feb 23;17(1):2470372. doi: 10.1080/19490976.2025.2470372 (PMC11853549; doi:10.1080/19490976.2025.2470372)
Supplement: Supplemental Material [file KGMI_A_2470372_SM5154.zip › Supplementary_Tables.docx]

| **Supplementary Table S1:** **The clinical features of PDAC patients in cohort 1 (n/%)** | | | |  |
| --- | --- | --- | --- | --- |
| Clinical | Total | no ED | ED |  |
| characteristics | (*n*=30) | (*n*=16, 53.3%) | (*n*=14, 46.7%) |  |
| **Gender, *n* (%)** |  |  |  |  |
| Male |  | 12(75.0) | 8(57.1) |  |
| Female |  | 4(25.0) | 6(42.9) |  |
| **Age, years, *n* (%)** |  |  |  |  |
| ≤65 |  | 9(56.3) | 5(35.7) |  |
| >65 |  | 7(43.7) | 9(64.3) |  |
| **PS, *n* (%)** |  |  |  |  |
| 0 |  | 10(62.5) | 7(50.0) |  |
| 1 |  | 6(37.5) | 7(50.0) |  |
| **Disease stage, *n* (%)** |  |  |  |  |
| I-II |  | 6(37.5) | 2(14.3) |  |
| III |  | 1(6.3) | 2(14.3) |  |
| IV |  | 9(56.3) | 10(71.4) |  |
| **Tumor location in pancreas, *n* (%)** |  |  |  |  |
| Head |  | 8(50.0) | 9(64.3) |  |
| Body or tail |  | 8(50.0) | 5(35.7) |  |
| **Metastases, *n* (%)** |  | 7(43.8) | 4(28.6) |  |
| No |  |  |  |  |
| Liver |  | 6(37.5) | 6(42.9) |  |
| Other organ sites |  | 3(18.7) | 4(28.6) |  |
| **Treatment, *n* (%)** |  |  |  |  |
| Adjuvant therapy |  | 7(43.8) | 2(14.3) |  |
| First-line therapy |  | 3(18.7) | 6(42.9) |  |
| Subsequent-line therapy |  | 6(37.5) | 6(42.9) |  |
|  |  |  |  |  |

| **Supplementary Table S2:** **The clinical features of PDAC patients in cohort 2 (n/%)** | | | | |  |
| --- | --- | --- | --- | --- | --- |
| Clinical | Total | low-*Lactobacillus* | | High-*Lactobacillus* |  |
| characteristics | (*n*=33) | (*n*=16, 48.5%) | (*n*=17, 51.5%) | |  |
| **Gender, *n* (%)** |  |  |  | |  |
| Male |  | 11(68.8) | 13(76.5) | |  |
| Female |  | 5(31.2) | 4(23.5) | |  |
| **Age, years, *n* (%)** |  |  |  | |  |
| ≤65 |  | 8(50.0) | 6(35.3) | |  |
| >65 |  | 8(50.0) | 11(64.7) | |  |
| **PS, *n* (%)** |  |  |  | |  |
| 0 |  | 9(56.3) | 11(64.7) | |  |
| 1 |  | 7(43.7) | 6(35.3) | |  |
| **Disease stage, *n* (%)** |  |  |  | |  |
| I-II |  | 2(12.5) | 5(29.4) | |  |
| III |  | 1(6.3) | 3(17.6) | |  |
| IV |  | 13(81.2) | 9(53.0) | |  |
| **Tumor location in pancreas, *n* (%)** |  |  |  | |  |
| Head |  | 8(50.0) | 9(53.0) | |  |
| Body or tail |  | 8(50.0) | 8(47.0) | |  |
| **Metastases, *n* (%)** |  | 3(18.8) | 8(47.0) | |  |
| No |  |  |  |  |  |
| Liver |  | 6(37.5) | 6(35.3) | |  |
| Other organ sites |  | 7(43.7) | 3(17.7) | |  |
| **Treatment, *n* (%)** |  |  |  | |  |
| Adjuvant therapy |  | 2(12.5) | 6(35.3) | |  |
| First-line therapy |  | 4(25.0) | 7(41.2) | |  |
| Subsequent-line therapy |  | 10(62.5) | 4(23.5) | |  |
|  |  |  |  | |  |
