# Supplementary material for: Upregulation of Lactobacillus spp. in gut microbiota as a novel mechanism for environmental eustress-induced anti-pancreatic cancer effects
Source: Gut Microbes. 2025 Feb 23;17(1):2470372. doi: 10.1080/19490976.2025.2470372 (PMC11853549; doi:10.1080/19490976.2025.2470372)
Supplement: Supplemental Material [file KGMI_A_2470372_SM5154.zip › Supplementary_Figures 241128.docx]

**Supplementary Figures**


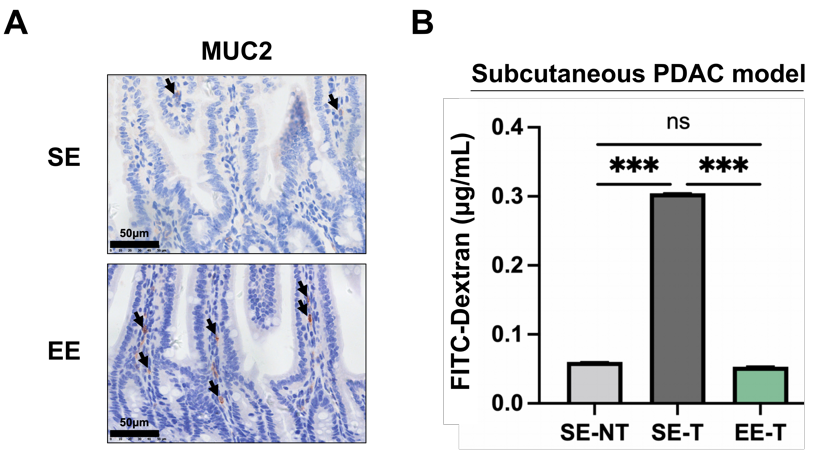


**Figure S1** Eustress restores gut microbiota diversity and strengthens the intestinal barrier in pancreatic cancer-bearing mice

(A) Compared with SE tumor-bearing mice, the EE mice showed an increased trend of MUC2 expression in intestinal epithelial evidenced by IHC staining of MUC2. Scale bar = 50 μm. (B) Quantitative analysis of FITC-dextran level in serum by permeability assay suggested that PDAC destroyed gut homeostasis and EE treatment partially restored the damage. ^***^, *p* < 0.001; ns, not significant.


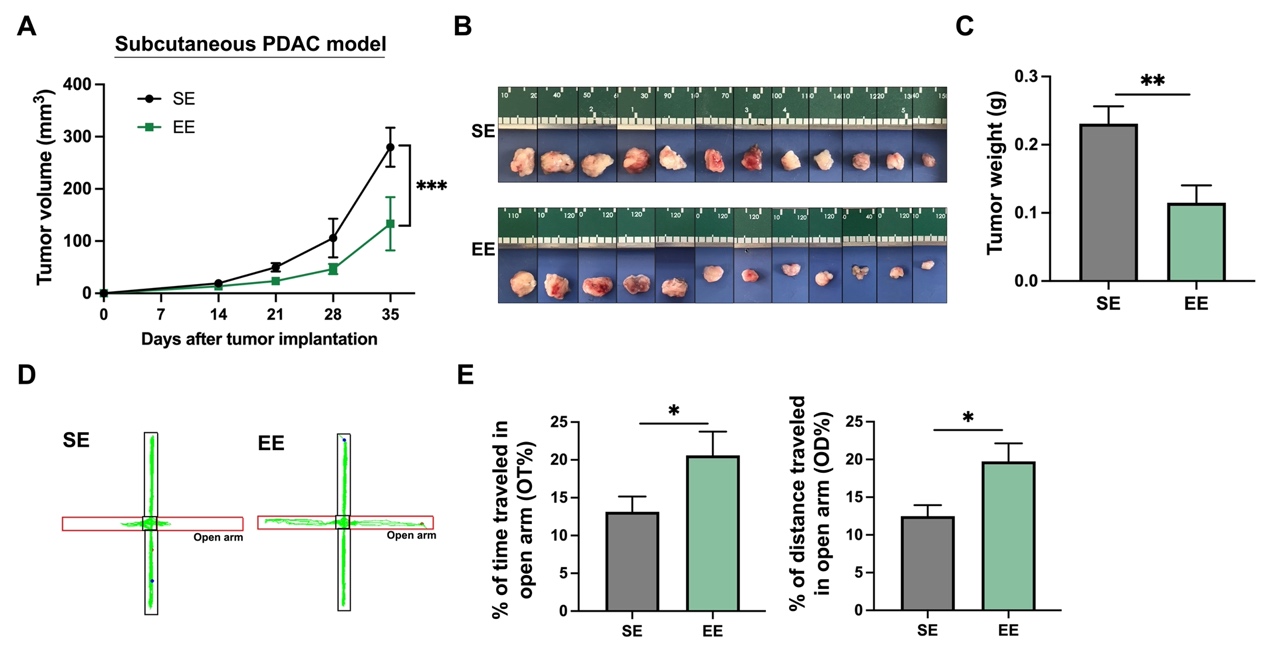


**Figure S2** EE mice showed reduced anxiety-like behaviors

EE housing largely suppressed the Panc02 tumor growth in the subcutaneous PDAC model, as evidenced by (A) tumor volume, (B) tumor images and (C) tumor weight (n=12/group). Compared with SE mice, EE mice showed reduced anxiety-like behaviors after 3 weeks of housing under EE conditions, as evidenced by (D) representative images and (E) the percentage of distance traveled in open arms and the percentage of time spent in open arms during the EPM test. ^***^, *p* < 0.001, ^**^, *p* < 0.01, ^*^, *p* < 0.05.


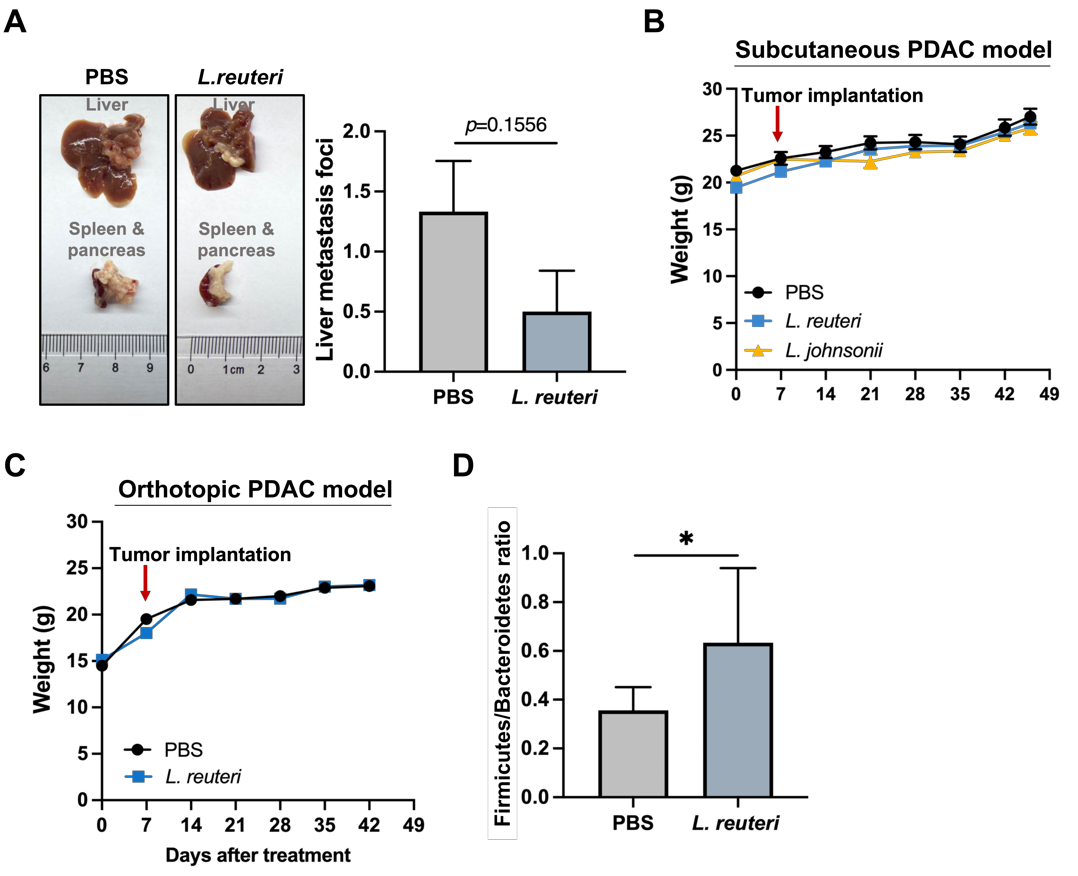


**Figure S3** *Lactobacillus spp.* inhibit pancreatic cancer growth

(A) The *L. reuteri*-treated mice with orthotopic pancreatic cancer exhibited a decreasing trend in hepatic metastasis, as demonstrated by (left) representative tissue images and (right) statistical analysis of liver metastasis foci. The administration of *L. reuteri* did not affect the body weight of tumor-bearing mice in both (B) the subcutaneous PDAC model and (C) the orthotopic PDAC model. (D) The administration of *L. reuteri* protects the gut barrier function in tumor-bearing mice, as evidenced by the increased Firmicutes/Bacteroidetes ratio using 16S rRNA sequencing analysis. ^*^, *p* < 0.05.


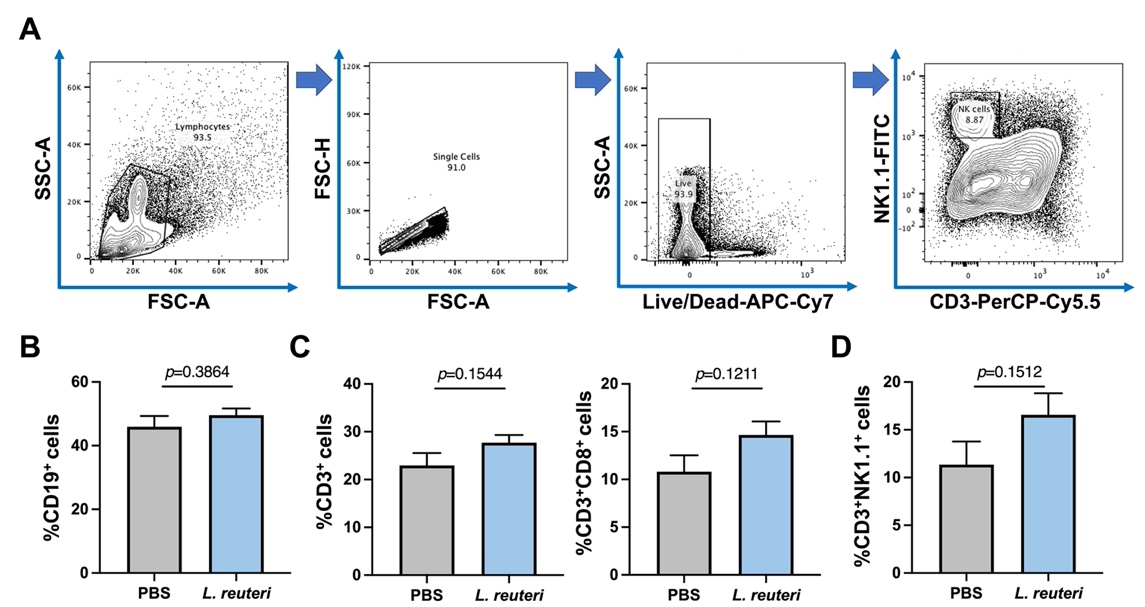


**Figure S4** NK cells were required for the anti-tumor effect of *L. reuteri*

(A) Representative gating strategy illustrating splenocyte subsets in mice with orthotopic pancreatic cancer. Flow cytometry results indicated that *L. reuteri* administration did not affect the percentage of (B) B cells (CD19^+^), (C) T cells (CD3^+^), CD8^+^T cells (CD3^+^CD8^+^) and (D) NKT cells (CD3^+^NK1.1^+^).
